# Best peer reviewers and the quality of peer review in biomedical journals

**DOI:** 10.3325/cmj.2012.53.386

**Published:** 2012-08

**Authors:** Armen Yuri Gasparyan, George D. Kitas

**Affiliations:** 1 Departments of Rheumatology and Research and Development, Dudley Group NHS Foundation Trust (A Teaching Trust of University of Birmingham, UK), Clinical Research Unit, Russell's Hall Hospital, Dudley, United Kingdom; 2 Arthritis Research UK Epidemiology Unit, University of Manchester, Manchester, UK

## Abstract

Current scholarly publications heavily rely on high quality peer review. Peer review, albeit imperfect, is aimed at improving science writing and editing. Evidence supporting peer review as a guarantor of the quality of biomedical publications is currently lacking. Its outcomes are largely dependent on the credentials of the reviewers. Several lines of evidence suggest that predictors of the best contributors to the process include affiliation to a good University and proper research training. Though the options to further improve peer review are currently limited, experts are in favor of formal education and courses on peer review for all contributors to this process. Long-term studies are warranted to assess the strengths and weaknesses of this approach.

Current scholarly publications visible in most prestigious indexing and citation tracking databases heavily rely on high-quality peer review (1,2). The whole process, albeit imperfect, is aimed at improving science writing, avoiding errors and methodological flaws in publications, and providing the readership with the best available scientific facts and appropriate interpretation (3,4). High-quality and evidence-based peer review in biomedical publications may ultimately contribute to the amendment of diagnostic and therapeutic guidelines and improve health outcomes. Peer review in biomedical scientific journals is widely seen as the virtue of science communication, and efforts are constantly taken to maintain its integrity (5).

The invention of peer review can be traced back to medieval times. However, most of its current attributes were proposed and nurtured in the past century by editors of *Science*, *Nature*, *Cell*, and some other top-tier general medical journals (6). Articles published in these journals have the greatest impact on science and practice. Each substantive manuscript undergoes rigorous internal and external review before appearing in the published form. Articles reporting results of a large trial, cohort study, systematic review, and meta-analysis are published after thorough evaluation by at least three experts and one statistician. Time and effort invested in the review of these articles are justified by their role in modernizing biomedical approaches and their applications.

Strict rules of peer review set by leading journals guide editors of other, smaller and newer journals in their relentless efforts to publish methodologically sound, original, and free of scientific misconduct articles (7). Rules are becoming even stricter as peer review and its adherence to good research reporting statements is becoming a matter of global concern (8,9).

Opponents of classical pre-publication peer review argue that the whole process is expensive, time-consuming, inconsistent, biased, and frequently abused (10). Another argument against the current system is that it is not powered enough to detect errors and it may unfairly diminish the value of even ground-breaking research and scientific ideas. Indeed, evidence supporting peer review as a guarantor of the quality of biomedical publications is currently lacking (11). A recent survey revealed poor knowledge of issues in peer review even among editors of high-ranking clinical medical journals (12).

Outcomes of peer review may vary, depending on the qualifications and number of reviewers (13). Scholarly journals differ in their publishing capacity, reviewer banks, and reviewer selection criteria for particular submissions. It is therefore not surprising that manuscripts rejected by one or many journals may eventually get published somewhere, even without responding to the comments raised by reviewers of rejecting journal(s) (14). In the modern-day publishing world there is an additional factor at play – the value of journal citation metrics, such as the *h* index and the 2-year journal impact factor (15). Reviewers and editors contributing to journals with high scientometric indicators are currently under pressure to select for publication potentially attractive and highly citable submissions, such as those on large clinical trials, cohort studies, and substantive reviews, leading to the rejection of articles on small studies and case reports, eventually appearing in lower-rank journals (16,17).

Editorial choice of the reviewers is determined by complex objective and subjective factors. There are still no universally accepted criteria of best reviewers, which may, at least partly, explain the differences in the quality within and between scholarly journals. A landmark study on reviewer comments found that the reviewers’ training in epidemiology or statistics, age below 60, residency in North America, and current involvement in research associate with high(er) quality comments (18). Another large survey of experienced biomedical reviewers identified only two critical factors of the quality: affiliation to a university hospital and young age (within ten post-graduate years) (19). Based on the analysis of the peer review in *Medicina Clinica* (Barcelona), it was also shown that adding a statistical reviewer substantially increases the quality of manuscripts (20), while additional reviews on adherence to current research reporting guidelines, such as CONSORT and STROBE, have only a slight effect (8).

In addition to the available evidence-based criteria of best reviewers, there are empirical, partly human factors determining the choice of reviewers and the reliance on their recommendations for publication. A large study on author- vs editor-suggested reviewers in a set of leading biomedical journals found no difference in the quality of the reviewer comments while it also advised against the blind reliance on the final recommendations made by author-suggested experts (21). Further studies reiterated these results and indicated that author-suggested reviewers tended to be more favorable in their recommendations for publication than author-excluded and editor-suggested experts (22,23). Another human-determined big issue is the so-called peer selection. The difficulties are particularly encountered when editors are to select reviewers for manuscripts written by either leading in their field authors or, on the other extreme, by young researchers (24). The issue is complicated further due to the limited number of reviewers with advanced skills, particularly in small scientific communities (eg, in medical ethics) (25) and in journals from non-mainstream science countries (26,27). Poor research environment and infrastructure, lack of adherence to high-standard editorial policies, restricted access to information sources, and communication difficulties with the reviewers impede the peer review in these countries (28,29). To use an extreme example, a recent survey of 245 Iranian scientific journals suggested that their editors struggle with external peer review and make decisions largely based on in-house comments (30).

The question arises as to whether there are options to tackle substandard and biased peer review? Training has long been viewed as a potentially useful option. Initial evidence on training points to the fact that short training packages may improve only some aspects of peer review, necessitating evaluation of longer-term interventions (31). Further evidence supports a positive role of manuals, practice reviews, and workshops for improving the quality of the review, at least in nursing research journals (32). A survey of 1675 reviewers for 41 nursing journals indicated that 65% of them expressed willingness to receive formal training, though only one-third of the reviewers managed to pass such a training (32).

Currently most professionals enhance their reviewer skills by learning from more experienced colleagues. In their attempt to improve peer review, some publishers facilitate this option by making reviewer comments open to the authors and reviewers, and by switching toward open-to-public peer review (33). Not surprisingly, most skilled reviewers are currently those with a good track record of own publications in high-quality journals and multiple reviewer contributions. The efficiency and implications of this approach, however, are hardly satisfactory, and therefore some experts support the need for formal education for all those who will be involved in science writing and reviewing (34). Furthermore, the prevailing expert opinion is in favor of incorporating relevant courses in the curricula of under- and post-graduate university education (35). The issue seems to be particularly important for non-mainstream science and non-Anglophone countries, where access to high-quality and well-polished English scientific literature is either limited or not on demand.

Traditional and newly launched learned associations may also take the lead in educating authors, reviewers, and editors alike. Fortunately, most of these associations are active in arranging conferences and short courses addressing a range of issues in science writing, editing, and indexing (9). It is hoped that more emphasis on strengths and pitfalls of peer review within the frames of these meetings will translate into proper guidance for all contributors of the peer review. Long-term studies are warranted to assess the efficacy of the educational approach to the improvement of peer review.

Acknowledgment The issues presented in the essay were discussed at a Continuous Professional Development (CPD) meeting at Clinical Education Centre of the Russell’s Hall Hospital, Dudley Group NHS Trust (A Teaching Trust of University of Birmingham), Dudley, UK. The authors thank all participants of the meeting for their comments and valuable suggestions.
